# Dextran-Based Edible Coatings to Prolong the Shelf Life of Blueberries

**DOI:** 10.3390/polym13234252

**Published:** 2021-12-04

**Authors:** Slađana Davidović, Miona Miljković, Milan Gordic, Gustavo Cabrera-Barjas, Aleksandra Nesic, Suzana Dimitrijević-Branković

**Affiliations:** 1 Faculty of Technology and Metallurgy, University of Belgrade, Karnegijeva 4, 11000 Belgrade, Serbia; mmiljkovic@tmf.bg.ac.rs (M.M.); suzana@tmf.bg.ac.rs (S.D.-B.); 2 Vinca Institute of Nuclear Sciences—National Institute of the Republic of Serbia, University of Belgrade, Mike Petrovića-Alasa 12-14, 11000 Belgrade, Serbia; milangordic@yahoo.com; 3 Unidad de Desarrollo Tecnológico, Universidad de Concepcion, Avda. Cordillera No. 2634, Parque Industrial Coronel, Coronel 4190000, Chile; g.cabrera@udt.cl

**Keywords:** dextran, edible films, food packaging

## Abstract

The development of edible films and coatings in the food packaging industry presents one of the modern strategies for protecting food products and ensuring their freshness and quality during their shelf lives. The application of microbial polysaccharides to the development of food package materials, as an alternative option to the commonly used plastic materials, is both economic and environmentally favorable. New edible films were developed using dextran from lactic acid bacterium *Leuconostoc mesenteroides* T3, and additionally plasticized by different concentrations of polyglycerol. The best tensile strength of the films was obtained using a formulation that contained 10 wt% of polyglycerol, which corresponded to a value of 4.6 MPa. The most flexible formulation, with elongation at break of 602%, was obtained with 30 wt% of polyglycerol. Water vapor permeability values of the films synthesized in this study were in the range of (3.45–8.81) ∗ 10^−12^ g/m s Pa. Such low values indicated that they could be efficient in preventing fruit from drying out during storage. Thus, the film formulations were used to coat blueberries in order to assess their quality during a storage time of 21 days at 8 °C. The results showed that dextran/polyglycerol films could be efficient in extending the shelf life of blueberries, which was evidenced by lower weight loss and total sugar solids values, as well as a delay in titratable acidity, in comparison to the uncoated blueberries.

## 1. Introduction

The maintenance of the quality of fresh food products represents a major challenge for the food industry. Many storage techniques have been developed in order to extend the shelf lives of food products, such as refrigeration at high humidity, modified atmosphere packaging or UV irradiation [1]. The use of edible films/coatings is another concept for food quality maintenance that has been receiving considerable attention [2,3,4,5]. Generally, an edible coating is a thin layer of edible material formed on the surface of food. Edible coatings can be applied and formed directly on the food by dipping, spraying, or brushing methods. By being edible and from renewable sources, edible coatings represent a unique category of packaging materials that differ from other conventional packaging materials. Various polysaccharides derived from plants (starch, pectin, cellulose derivatives) [6,7,8,9] and marine waste (chitosan, alginate, carrageenan) [10,11,12,13,14,15] have been investigated as edible films for the potential preservation of fruit. Polysaccharides are generally attractive natural polymers to be used as edible protective coating on fruit due to their good oxygen and CO_2_ barriers. However, their poor mechanical properties and water vapor barrier, as a consequence of their highly hydrophilic nature, limits their practical application in the food-packaging sector. Hence, the selection of new polysaccharides and their modifications have been intensively studied in order to overcome the main drawbacks of polysaccharide-based films and coatings.

Polysaccharides produced by microorganisms have gained increased attention, since they showed good film-forming properties, as well as adhesive properties [16,17]. Moreover, from a technological point of view, microbial polysaccharides are easier to produce compared to plant or marine polysaccharides, since fermentation processes are controllable independently of climate conditions and usually last for few days. The extraction and purification of the final product from the fermentation medium is also relatively simple. Abundant microorganisms that produce polysaccharides represent an inexhaustible source of biopolymers with diverse structures and physicochemical properties that are widely exploited in various industries, such as medicine, pharmacy, cosmetics, agriculture, etc. In the food industry, microbial polysaccharides are used as thickeners, stabilizers, and emulsifiers.

Dextran is an exopolysaccharide produced by different microbial strains, mostly of the genera *Leuconostoc*, *Lactobacillus,* and *Streptococcus,* when grow on the sucrose-rich media. Dextran is composed of α-(1,6)-linked d-glucopyranose residues and α-(1→2), α-(1→3), α-(1→4) branched linkages [18]. The type of strain influences the degree of branching and physical properties of the obtained dextran. Due to its versatile properties, dextran has found applications the in pharmaceutical industry as a blood plasma volume expander [19]. In the food industry, dextran is used as thickener for jams and ice creams since it prevents the crystallization of sugar, improves moisture retention, and maintains the flavor of food products [20,21]. 

Since dextran is a tasteless, food-grade compound with excellent biocompatibility and non-toxicity, it can potentially be used as a new food packaging material. It was hypothesized that dextran could act as an oxygen scavenger and moisture-resistant additive [22], and, along with a good water vapor barrier [23], it might be a good choice of biopolymer for edible coatings and potentially provide longer shelf lives of fruit, than the polysaccharides investigated so far. 

Generally, all polysaccharide-based films require the incorporation of a plasticizer to reduce their brittleness and to improve the flexibility, toughness, and tear resistance of the material. The most frequently used plasticizers are polyols. In this work, polyglycerol has been chosen as a plasticizer, due to its high biocompatibility and specific molecular structure. Namely, polyglycerols belong to a class of multihydroxy-functional glycerol molecules bonded together by ether linkages. It was shown that the presence of longer molecular chains of the plasticizer could provide stronger intermolecular bonds with the biopolymer, which could result in better mechanical and/or water vapor barrier performances in biopolymer-based films [24,25]. 

Based on the constant necessity of the food industry for new and innovative products, dextran produced in our lab by *Leuconostoc mesenteroides* T3, isolated from kefir grains, was used as a raw material to the develop edible films. Hence, the aim of this study was to evaluate the potential of dextran as a microbial polysaccharide to be applied as a sustainable and edible bio-coating for blueberries. The blueberries were chosen as model system of fruit due to their susceptibility to water loss. Considering the health benefits of blueberries, the longer shelf life achieved by an edible coating could be a positive step for consumers and the industry. 

The research objective of this work was to test the mechanical, thermal, and water vapor barrier properties of dextran–polyglycerol films and to assess the ability of the coatings to prolong the shelf life of blueberries, by monitoring their weight loss, titratable acidity, and solid content within the targeted storage time. To the best of our knowledge, this paper is the first report about dextran-based edible films with an examination of their influence on the shelf lives of food products. 

## 2. Results and Discussion

### 2.1. Mechanical Analysis

Tensile strength (TS, MPa) indicates the maximum tensile stress that a film can sustain. Elongation at break (ε, %) is the maximum change in the length of a test sample before breaking. The effect of polyglycerol concentration on the mechanical properties of dextran-based films is presented in Table 1. The mechanical properties of neat dextran films without polyglycerol have not been performed, since the film was brittle and broke into pieces during the drying process. The increasing polyglycerol content in the dextran matrix reduced the tensile strength of the films from 4.60 MPa (10% of PG) to 0.19 MPa (30% of PG) and significantly increased elongation at break (from 123% to 602%). This trend can be explained by an increased spatial distance between polymer chains with the addition of polyglycerol [8,23,26]. Polyglycerol acted as a plasticizer and increased the mobility of the polymer chains, which contributed to more stretchable and flexible films. The TS values were comparable with the ones reported for other polysaccharide films such as pectin (7.20 MPa) [27], chitosan (12.6 MPa) [28], agar (0.39 MPa) [29], starch (2.03 MPa) [30], and pullulan (4.30 MPa) [31]. It is important to highlight that the addition of polyglycerol into the dextran matrix led to films with significantly improved flexibility (100–600%), since the elongation at break of polysaccharide-plasticized films generally range between 10 to 70% [32,33,34]. Although the elongation at break of D/PG30 film was 602%, the tensile strength of this film did not meet the criteria for food packaging material. The food packaging material should have a tensile strength above 4 MPa and elongation at break above 15% [35]. Hence, the D/PG10 was the only suitable material that satisfied the requirements for the mechanical properties for it to be potentially applied in food packaging sector.

### 2.2. Water Vapor Permeability Analysis

One of the key roles of edible films is to control moisture transfer between the food and surrounding atmosphere. Therefore, the WVP value should be as low as possible. The WVP is significantly influenced by the content of a plasticizer, the origin of the biopolymer, and the thickness of the edible films. As it is shown in Table 1, WVP values of dextran-plasticized films were in the range between 3.45 × 10^−12^ and 8.81 × 10^−12^ g/m s Pa. As polyglycerol concentration increased, the permeation of water vapor molecules through the films increased as well. The increase in WVP was attributed to the structural reorganization of the dextran network and the increase in the free volume and segmental motions, which, as a result, allowed easier diffusion of water molecules through the film. It is important to underline that the WVP values of dextran–polyglycerol films were 1–3 orders of magnitude lower than other polysaccharide films reported in the literature. For example, pectin-based films had WVP values of 10^−10^ g/m s Pa [8,27], agar films had WVP values of 10^−9^ g/m s Pa [36], and chitosan films had WVP values of 10^−10^ g/m s Pa [37]. The obtained low WVP values of dextran-based films suggested that dextran was a good choice of polysaccharide that could provide efficient water retention when applied as a coating to fresh fruits.

### 2.3. Thermogravimetric Analysis

**Figure 1:**
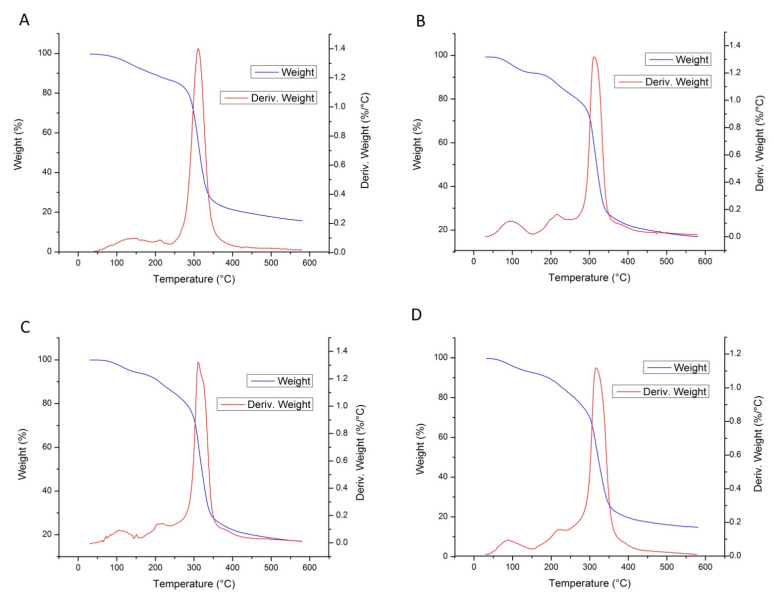
TG/DTG curves of neat dextran (**A**) and dextran films with 10% (**B**), 20% (**C**), and 30% polyglycerol (**D**).

To assess the effect of polyglycerol addition on the thermal decomposition behavior of dextran-based films, non-isothermal thermogravimetry of neat dextran and edible films was performed. The TG/DTG curves are shown in Figure 1. This property is important from the technological aspect, considering that high temperatures are often applied during the synthesis and processing of materials. In all samples three degradation steps can be observed. The first degradation step occurred up to 110 °C and it is related to the evaporation of moisture/free water. The second degradation step that occurred in the range between 200 °C and 250 °C was associated to depolymerization and redistribution in the position of linkages as a result of the hydrolytic reaction, where dextrin of low viscosity under air atmosphere was formed [38]. The weight loss in this degradation step was around 20% for all samples. The third step that occurred in the range between 250 °C and 350 °C was described as the degradation of main dextran chains [39], with approximate weight loss of 75%. As it can be noticed, the addition of polyglycerol into the dextran matrix did not influence significantly the thermal stability of films, since there were no changes in T_onset_ temperatures. The total mass lost at 600 °C for all films was approximately 80–85%. 

A similar degradation pattern was obtained for other dextran materials [39,40]. Moreover, according to data in the literature, plasticizer does not significantly influence the thermal stability of polysaccharide films, and the thermal stability of dextran films presented in this work were in the same range as most commonly used polysaccharides, such as pectin, alginate, and chitosan [24,26].

### 2.4. Quality of Blueberries

**Figure 2:**
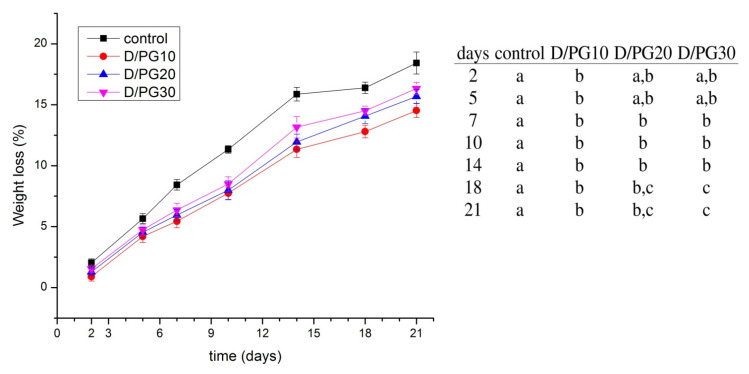
Weight loss (%) of uncoated (control) and coated blueberries during 21 days of storage at 8 °C. Data are the average of 3 replicates ± standard deviation. Different letters within rows indicate significant differences between experimental data according to Tukey’s test (*p* < 0.05).

The weight loss of fruit is an important factor for predicting the quality of fruit. It usually happens due to the migration of water from fruit to the environment. The weight losses of uncoated and coated blueberries during storage of 21 days at 8 °C are shown in Figure 2. A change in weight loss was observed for all blueberry samples. It has been stated that cold storage conditions could cause a difference in vapor pressure between the blueberries and the environment, which as a result leads to slight weight losses [41]. After the storage time of 21 days, the uncoated blueberries showed the highest weight loss (18.4 ± 0.9%), while the samples coated with D/PG10 formulation exhibited the lowest changes (14.5 ± 0.6%). An increase in plasticizer content led to greater weight loss of the blueberries. However, these changes were significant only between the D/PG10 and D/PG30 coated samples (14.5 ± 0.6% and 16.3 ± 0.5%, respectively). Since polyglycerol interacts with water molecules, its higher content in film leads to slightly greater losses. Mannozzi et al. observed no difference in the weight loss between pectin/glycerol, alginate/glycerol, and pectin/alginate/glycerol coated and uncoated blueberries after 14 days of storage at 4 °C (difference below 5%) [7]. The authors obtained the same results when they coated blueberries with chitosan/procyanidin formulation [1]. On the other side, Verieria et al. demonstrated that chitosan/aloe vera coating could significantly delay the weight loss of blueberries (3.7%), in comparison to the uncoated blueberries (6.2%) during storage time of 25 days at 5 °C [15]. However, chitosan without an additional antimicrobial additive did not provide significant changes in the weight loss of blueberries, with respect to the control samples. The weight loss of blueberries (12.7%) decreased after coating them with alginate/cyclolipopeptide formulations (4.4%) [42]. In this work, dextran-based coatings minimized the weight loss of blueberries by 11–21%, in comparison to the uncoated samples. These results were expected since the WVP results for dextran/PG films proved their excellent barrier properties, thus preventing the migration of water from the blueberries. Although some authors obtained very low weight loss of coated blueberries (around 4–5%), these coatings always contained an antimicrobial additive incorporated in the polysaccharide matrix. 

**Figure 3:**
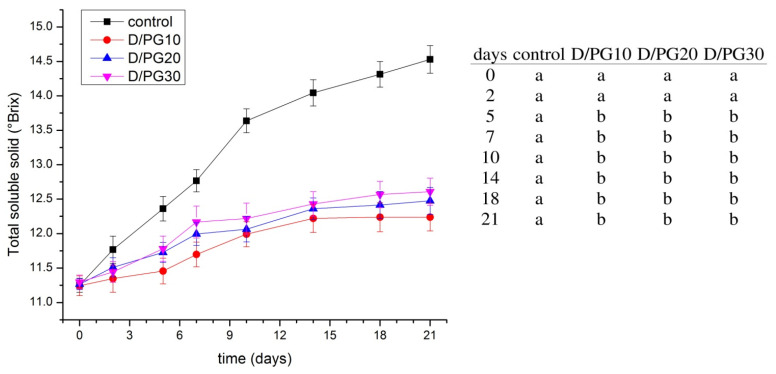
Total soluble solid content of uncoated (control) and coated blueberries during 21 days of storage at 8 °C. Data are the average of 3 replicates ± standard deviation. Different letters within rows indicate significant differences between experimental data according to Tukey’s test (*p* < 0.05).

Total soluble solid content (TSS, °Brix) is an important maturity index for fruit, and edible coatings are effective in lowering TSS, or, in other words, lowering ripening rates. As shown in Figure 3, TSS values increased during storage time for uncoated and all coated blueberries. The increase in TSS is a result of the hydrolysis of carbohydrates that produce total soluble sugars such as sucrose, glucose, and fructose. TSS appeared to be higher for uncoated blueberries than for coated ones, which confirmed the efficiency of coating to reduce the rate of respiration of the blueberries and hence to lessen the hydrolysis of carbohydrates. The TSS of the control sample increased from 11.2 to 14 on day 14, and maintained near to that value until day 21 of storage at 8 °C. In the case of blueberries coated by D/PG10, TSS increased from 11.2 to 12.2 within 21 days. On the other hand, a plasticizer content in the coating formulation did not show significant influence on changes in TSS values among different coated samples (which ranged between 12.2 and 12.6 °Brix after 21 days). This trend and values of the coated blueberries compared to the uncoated blueberries were in agreement with the data in the literature [15].

**Figure 4:**
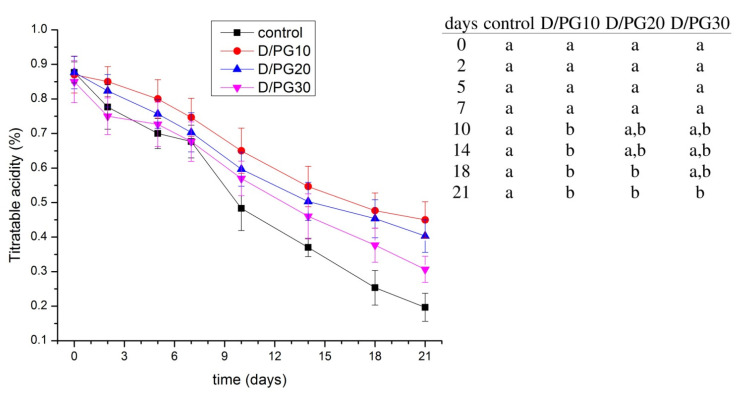
Titratable acidity of uncoated (control) and coated blueberries during 21 days of storage at 8 °C. Data are the average of 3 replicates ± standard deviation. Different letters within rows indicate significant differences between experimental data according to Tukey’s test (*p* < 0.05).

The effect of coatings on titratable acidity of blueberries is presented in Figure 4. In all cases, titratable acidity decreased within the time. Moreover, TA values were higher for all coated samples (the decline was approximately from 0.9 to 0.31%) than uncoated ones (the decline was from 0.9 to 0.2%). The samples coated with D/PG10 showed the least decrease in titratable acidity (from 0.9 to 0.45%). The increased PG concentration in the films provided a decrease in TA values, however, these differences were not significant (*p* < 0.05). The obtained decrease in TA within the time was the result of a change in the prevalent citric acid in blueberries, which acted as a substrate for many enzyme -catalyzed reactions during aerobic respiration in the plant cells, thus making the fruits taste relatively sweeter [10]. A decline in acidity demonstrated the acceleration of maturity (ripening). Since a lower decline in TA was observed for coated blueberries, dextran–PG formulated coatings were shown to be efficient in the delay of ripening. Data in the literature show that the ripening process was affected by the coatings’ composition. According to the results reported by Chiabrando and Giacalone [43], blueberries coated with alginate and chitosan retained high values of TA during 45 days of cold storage. The same study showed that alginate, as a sole coating, had an opposite effect, i.e., the coating enhanced the ripening of blueberries. Accelerated maturation of blueberries was also noticed with a coating based on a quinoa protein/chitosan/sunflower oil [10]. 

Overall, it was shown that dextran produced in our lab using lactic acid bacterium *Leuconostoc mesenteroides* T3, isolated from water kefir grain, could be good source of raw material for the preparation of edible coatings for blueberries. The dextran–polyglycerol coating on blueberries provided lower weight loss and TAA in comparison to the uncoated fruit, thus delaying their dehydration. However, large-scale production of dextran in a controlled manner still needs to be investigated in order to verify the scalability and quality of the produced biopolymer. Moreover, the controlled application of edible coating onto fruits should be more studied, in order to achieve full surface coverage of the fruit. Nevertheless, the microbial quality of blueberries could be affected in the long term if only biopolymers based on sugars, without additives, were used as a coating. Hence, future directions should address the influence of antimicrobials and polyphenols in dextran–polyglycerol films to further minimize the weight loss of blueberries and prevent microbial contamination.

## 3. Materials and Methods

The dextran was obtained by dextransucrase from *Leuconostoc mesenteroides* T3, a natural isolate from water kefir grain, as previously described [26]. Polyglycerol (PG), a mixture containing a minimum of 85% diglycerol, triglycerol, and tetraglycerol with only trace amounts of glycerol, was purchased from Solvay. Tween80, NaOH, and HCl were purchased from Sigma Aldrich. All chemicals were used as they were received, without further purification. Organic blueberries (Highbush Duke), harvested in June 2016, were purchased from a local market and stored. Fresh blueberries of a similar color, maturity degree, and no visible skin damage were selected for the experiments.

### 3.1. Preparation of Dextran Films

All films were prepared using a solvent casting method. First, 2 wt% of dextran solutions were prepared by dissolution of 1 g of dextran in 50 mL of distilled water at 70 °C. A certain amount of a plasticizer and Tween80 was added to each dextran solution. The amount of plasticizer varied from 10–30 wt% by mass of dextran, whereas the concentration of Tween80 was kept constant at 5 wt%. The mixtures were stirred until the homogeneity was established. Dextran-based solutions were cast in round-shaped Teflon molds (11 cm in diameter) and dried in an oven at 30 °C for 72 h.

### 3.2. Characterization

#### 3.2.1. Mechanical Analysis

Mechanical properties (tensile strength, TS, and percentage of elongation at break, E) were determined using an Instron M 5543 universal testing machine. Crosshead speed was 2 mm min^−1^ for testing samples. Six film specimens (mold 5 mm × 30 mm) of each sample were tested. The average thickness of all samples was in the range of 90–110 µm, depending on the concentration of plasticizer in the film. The reported results were presented as average values of six measurements.

#### 3.2.2. Thermal Analysis

Thermogravimetric analysis was performed on a Mettler Thermogravimeter Analyzer Mod. TG 50 (Mettler Toledo Corp., Zurich, Switzerland). The measurements were performed on samples of about 10 mg, placed in ceramic crucibles, and heated from room temperature to 600 °C at a heating rate of 10 °C/min, at air atmosphere. 

#### 3.2.3. Water Vapor Permeability

The water vapor permeability (WVP) through the composite films was determined gravimetrically according to the ASTM E96 standard. Films were sealed in a 35 mm circular opening of a steel permeation cell containing distilled water (~100% relative humidity inside the cell). The permeation cell was kept in a chamber with controlled relative humidity of 50%. The weight of the permeation cell was measured every 2 h until the weight was constant. The WVP of the films were calculated using the following equation:

WVP = (ΔG × l)/(t × A × Δp)

where ΔG was the weight change (g), t was the time during which ΔG occurred (h), A was the test area cup (m^2^), l (m) was the thickness of the film, and Δp was the water pressure difference between both sides of the film (Pa). Measurements were performed in triplicate and average data were used for calculations. 

#### 3.2.4. Coating of Blueberry Samples

Blueberries were dipped in the coating solutions for 30 s and left to dry in a ventilated oven at 30 °C. Blueberries dipped in distilled water with the same procedures were used as a control. The coated berry samples were then placed in plastic trays (PET) and stored at 8 °C for 21 days. Three trays for every sampling time were made, containing 30 blueberries each, from which fruits were taken randomly from the three trays and used for analytical determinations.

#### 3.2.5. Quality of Blueberries

The weight loss of uncoated and coated blueberries was determined periodically by weighing the samples with a digital balance and it was reported as a percentage loss in weight of samples, based on the initial weight. The average of the three measurements was accepted. The change in weight of the blueberries was calculated according to the following equation:

W_L_(%) = (W_0_ − W_1_)/W_0_ × 100

where W_0_ and W_1_ were the accurate dried weight before and after being kept at 8 °C respectively.

Soluble solid content analysis was performed at 25 °C by measuring the refractive index of the blueberry juice with a hand refractometer. Results were expressed as °Brix.

The titratable acidity (TA) was followed according to the following procedure: ten grams of blueberries from each sample group were ground in a blender; six grams of blueberry juice was homogenized with 50 mL of distilled water; a few drops of phenolphthalein were added and the mixture was titrated with 0.1 M NaOH; the point neutrality was reached when the indicator changed color from colorless to pink; results were expressed as % (grams of citric acid equivalent per 100 g of blueberry); the average of the three measurements was accepted; the titratable acidity was calculated according to the following equation.

TA (%) = V(NaOH) × C(NaOH) × 0.064 × 100%/m

where m (g) is the accurate weight of the blueberry juice.

#### 3.2.6. Statistical Analysis

The presented results are mean values of independent experiments with ± standard deviations. One-way analysis of variance (ANOVA) followed by Tukey’s test was used for the comparison of mean values. Differences were considered significant at *p* < 0.05. Data analysis was performed using OriginPro 8.5.

## 4. Conclusions

This study presented the mechanical, thermal, and water vapor barrier properties of novel dextran films plasticized with different concentrations of polyglycerol. The presented results showed that increasing the concentration of polyglycerol from 10 to 30 wt% led to a significant drop in tensile strength and an increase in the films’ elasticity, while thermal stability was not significantly affected by the plasticizer content in the film. The film formulation that best met the criteria for food package material was obtained with 10 wt% polyglycerol. The elongation at break of this film formulation was 122.5% and tensile strength was 4.6 MPa. All the films examined in this study exhibited outstanding barrier properties with noticeably low values for water vapor permeability. The effectiveness of dextran/polyglycerol coatings to preserve the fresh fruit during their shelf lives was proved using blueberries. Namely, the weight loss of blueberries coated by D/PG10 was delayed by 21%, the titratable acidity declined by 56% and total sugar solids increased by 16 %, in comparison to the uncoated blueberries. Overall, the results of the study suggested that microbial polysaccharide dextran has excellent potential for the development of new food package materials.

## Figures and Tables

**Table 1:** Effect of polyglicerol content on the physico–chemical properties of dextran-based films.

Sample	TS, MPa	ε, %	WVP × 10^12^, g/m s Pa
D/PG10	4.60 ± 0.23 ^a^	122.5 ± 9.5 ^a^	3.45 ± 0.23 ^a^
D/PG20	1.30 ± 0.15 ^b^	265 ± 24 ^b^	5.78 ± 0.46 ^b^
D/PG30	0.19 ± 0.01 ^c^	602 ± 58 ^c^	8.81 ± 0.78 ^c^

^a,b,c^ Different letters within columns indicate significant differences between experimental data according to Tukey’s test (*p* < 0.05). Tensile strength (TS) and elongation at break (ε) data are the average of 6 replicates ± standard deviation. Water vapor permeability (WVP) data are the average of 3 replicates ± standard deviation.

## Data Availability

Not applicable.
